# Green Light-Emitting Devices Based on Perovskite CsPbBr_3_ Quantum Dots

**DOI:** 10.3389/fchem.2018.00381

**Published:** 2018-08-28

**Authors:** Han Yu, Guimin Tian, Weiwei Xu, Shengwei Wang, Huaikang Zhang, Jinzhong Niu, Xia Chen

**Affiliations:** ^1^College of Agriculture, Jilin Agricultural University, Changchun, China; ^2^College of Science, Henan Institute of Engineering, Zhengzhou, China; ^3^National & Local United Engineering Laboratory for Chinese Herbal Medicine Breeding and Cultivation, School of Life Sciences, Jilin University, Changchun, China

**Keywords:** CsPbBr_3_, perovskite, quantum dots, light-emitting devices, high performance

## Abstract

In this paper, high quality green-emitting CsPbBr_3_ quantum dots (QDs) are successfully synthesized by hot-injection method. Different injection temperatures are tested to optimize the synthesis conditions. High brightness with the photoluminescence (PL) quantum yields (QYs) up to 90% and narrow size-distribution with the full width at half-maximum (FWHM) of 18.5 nm are obtained under the optimized conditions. Green light emitting diodes (LEDs) based on the CsPbBr_3_ QDs are successfully demonstrated by combining solution method with vapor deposition method. Composite films of poly[9,9-dioctylfluorene-co- N-[4-(3-methylpropyl)]-diphenylamine] (TFB) and bathocuproine (BCP) layers are chosen as the hole-transporting and the electron-transporting layers, respectively. The highly bright green QD-based light-emitting devices (QLEDs) showing maximum luminance up to 46,000 cd/m^2^ with a low turn on voltage of 2.3 V, and peak external quantum efficiency (EQE) of 5.7%, corresponding to 19.9 cd/A in luminance efficiency. These devices also show high color purity for electroluminescence (EL) with FWHM <20 nm, and no redshift and broadening with increasing voltage as well as a spectral match between PL and EL.

## Introduction

Quantum dots based light-emitting devices (QLEDs) are very attractive to industry and academia for potential applications in high color rendering index (CRI) solid-state lighting and high color saturation displays due to their tunable emission range, narrow emission linewidth, and high photoluminescence (PL) efficiencies throughout the visible light region (Shirasaki et al., 2013; Tan et al., 2014). Inorganic perovskite quantum dots (QDs) not only have the advantages of conventional inorganic QDs, such as high photoluminescence quantum yields (QYs), wide wavelength tunability (wavelengths range from ultraviolet light through visible light to near-infrared light, 400–800 nm), very high color purity [full width at half maximum (FWHM) <20 nm], but also show low material cost, simply tunable band gap, with a reasonable ionization energy (IE), and easy to scale up synthesis which are incomparable over traditional QDs (Tan et al., 2014; Protesescu et al., 2015; Song et al., 2015; Wang et al., 2015; Colella et al., 2016). Hot injection method and room temperature synthesis method are usually used for the synthesis of inorganic perovskite QDs. Kovalenko and co-workers and Li and co-workers have synthesized CsPbX_3_ (X = Cl, Br, I) inorganic perovskite QDs, which exhibited QYs as high as 90%, narrow line width and high stability, which make this all-inorganic perovskite family CsPbX_3_ (X = Cl, Br, I) promising for both optical and optoelectronic applications (Nedelcu et al., 2015; Li et al., 2016). Shortly after that, low threshold, color and shape tunable amplified stimulated emission was reported by other groups simultaneously by using hot injection method (Protesescu et al., 2016; Li Q. et al., 2017). In terms of perovskite based light-emitting devices, external quantum efficiency (EQE) of green perovskite light-emitting diodes (PeLEDs) is notably improved to 14.36% via composition, phase engineering and surface passivation effect that is achieved by coating organic small molecule trioctylphosphine oxide (TOPO) on the perovskite film surface (Yang et al., 2018). And the current efficiency of PeLEDs is improved from 0.43 to 45.4 cd/A (Song et al., 2018). Although the inorganic perovskite QDs possess excellent fluorescence properties as organic perovskite and have a higher stability compared with organic perovskite (Protesescu et al., 2015), for light-emitting devices based on inorganic perovskite QDs, efficiency and brightness are still low and urgent needed to improve (Yantara et al., 2015; Wei et al., 2016; Cho et al., 2017; Wu et al., 2017; Yu et al., 2017). The lower device performance of inorganic perovskite QLEDs are mainly attributable to the following points. Firstly, facile thermal ionization of excitons with low binding energy generated in the perovskite layer results substantial luminescence quenching in CsPbBr_3_ based QLEDs. Secondly, low film quality of CsPbBr_3_ QDs. When using the traditional spin-coating method for the preparation of inorganic perovskite film, in the process of heating to remove the solvent and surfactant, many pinholes, cuboids of large grain size, and crack are created, such film is easy to cause leakage current and unable to meet the requirements of high performance devices. Therefore, improve the efficiency and brightness of inorganic perovskite QLEDs need to limit the large exciton diffusion length of excitons or charge carriers and reduce the possibility of exciton dissociation into carriers, and improve the flatness and uniformity of inorganic perovskite emitting layer (Cho et al., 2015). The uniform morphology of a perovskite film is one of important factor in realizing PeLEDs with high efficiency and full-coverage electroluminescence. A uniform and continuous morphology of perovskite film also affecting the subsequent deposition of functional layers with solution procedures (Bade et al., 2015, 2017; Li G. et al., 2015; Li J. et al., 2015).

Herein, high quality green-emitting CsPbBr_3_ quantum dots (QDs) are successfully synthesized by injecting PbBr_2_ precursors into Cs precursor solution. The effects of different injection temperatures are investigated to optimize the synthesis conditions. High brightness with the PL QYs up to 90% and narrow size-distribution with the full width at half-maximum (FWHM) below 18.5 nm are obtained. Green light emitting diodes (LEDs) based on the CsPbBr_3_ QDs are fabricated by combining solution method with vapor deposition method. Composite films of poly[9,9-dioctylfluorene-co-N-[4-(3-methylpropyl)]-diphenylamine] (TFB) and bathocuproine (BCP) layers are chosen as the hole-transporting and the electron-transporting layers, respectively. Highly bright green CsPbBr_3_ QLEDs showing maximum luminance up to 46,000 cd/m^2^ with a low turn on voltage of 2.3 V, and peak external quantum efficiency (EQE) of 5.7%, corresponding to 19.9 cd/A in luminance efficiency. We attribute the improvement of device performance to the uniform and continuous morphology of perovskite film, suppression of self-absorption and Förster resonance energy transfer (FRET). These devices also show high color purity for electroluminescence (EL) with FWHM <20 nm, no redshift and broadening with increasing voltage as well as a spectral match between PL and EL.

## Materials and methods

### Chemicals

All reagents were used as received without further experimental purification. Cesium carbonate (Cs_2_CO_3_, 99.9%), oleic acid (OA, 90%), 1-octadecene (ODE, 90%), oleylamine (OAm, 80–90%), lead bromide (PbBr_2_, ABCR, 98%), toluene (99%), and chlorobenzene (99%) were purchased from Aldrich. Hexanes (analytical grade) and methanol (analytical grade) were obtained from Beijing Chemical Reagent Ltd., China.

### Synthesis methods

#### Preparation of precursor solution

PbBr_2_ (0.5505 g, 1.5 mmol, Aldrich, 99.9%) was loaded into 50 mL three-neck flask along with octadecene (5 mL, Sigma-Aldrich, 90%), oleic acid (5 mL, OA, Sigma-Aldrich, 90%), and oleylamine (5 mL, OAm, 80–90%), maintained for 15 min at 120°C under N_2_ flow, the reaction mixture was cooled to 80°C before injection.

#### Synthesis of CsPbBr_3_ QDs

Cs_2_CO_3_ (0.0204 g), octadecene (10 mL, Sigma-Aldrich, 90%), and oleic acid (0.5 mL, OA, Sigma-Aldrich, 90%) were loaded into 25 mL three-neck flask and dried under N_2_ for 15 min at 120°C. After complete solubilization of Cs_2_CO_3_, the temperature was raised to 140–200°C (for tuning the QD size) and PbBr_2_ solution (2 mL, prepared as described above) was quickly injected and, the reaction mixture was cooled by the ice-water bath at once.

### Fabrication and characterization of QLEDs

The QLEDs were fabricated on ITO pre-patterned glass substrates with a sheet resistance of 20 /sq. The substrates were sonicated sequentially in detergent, deionized water, acetone, and isopropanol for 15 min, respectively, followed by treating with UV-Ozone for 15 min in air. The PEDOT:PSS aqueous solution was deposited as hole injection layer (HIL) via spin-coating onto the top of ITO layer and annealed at 140°C for 15 min in air, then transferred to N_2_-filled glovebox rapidly. Poly(9,9-dioctylfluorene-co-N-(4-butylphenyl)diphenylamine (TFB) was dissolved in chlorobenzene with a concentration of 8 mg/mL and deposited onto the PEDOT:PSS layer (3,000 rpm for 30 s) as hole transporting layer (HTL), and then annealed at 150°C for 30 min. In sequence, perovskite CsPbBr_3_ QDs (30 mg/mL in hexane) were spin-coated onto the TFB layer at 2,000, 2,500, 3,000, and 4,000 rpm for different thickness of QDs layer. Finally, CBP (40 nm), LiF (1 nm), and Al (100 nm) was deposited using thermal evaporation through shadow masks at rate of 2.0, 0.2, and 3.0 Å/s, respectively, under a high vacuum (<8 × 10 ^−7^ Torr). The devices were encapsulated then with a cover glass fixed with UV-curable resins.

### Characterization

X-ray diffraction (XRD) studies of products were carried out with a Bruker D8 advance X-ray diffractometer using Cu Kα radiation (wavelength = 0.154 nm). The transmission electron microscope (TEM) images were obtained using a JEOL JEM-2100 electron microscope operating at 200 kV. Room temperature UV-vis absorption spectrum was characterized with an ultraviolet visible near infrared spectrophotometer (mode Shimadzu UV3600). PL spectrum was measured with a fluorescence spectrometer (mode Agilent Cary Eclipse). PL quantum yields (QYs) were measured using an absolute PL QY measurement system (FLSP920) in an integrating sphere. Transient PL measurements were carried out using Edinburgh Instruments FL920 Spectrometer and the wavelength (hydrogen lamp as the excitation source) was 405 nm. X-ray photoelectron spectrum (XPS) measurements were performed on an AXIS ULTRA X-ray photoelectron spectroscope, using Al Kα radiation with an anode voltage of 15 kV and emission current of 3 mA. Fourier transform infrared (FTIR) spectra were recorded on a Nicolet6700 spectrometer. The surface morphology of CsPbBr_3_ layer in LED was characterized by using atomic force microscope (AFM, the Dimension FastScan, Bruker). The current density–voltage–luminance characteristics and EL spectra of the QLEDs were measured by a programmable Keithley model 2400 power supply and a Photo-research PR735 spectrometer at room temperature in air. An Ocean Optic UV-vis-NIR spectrophotometer was used to study the electroluminescence (EL) spectrum. The luminance and current efficiency were then calculated from the known portion of the forward emission and the LEDs output spectra. All the measurements were performed under ambient conditions.

## Results and discussion

**Figure 1:**
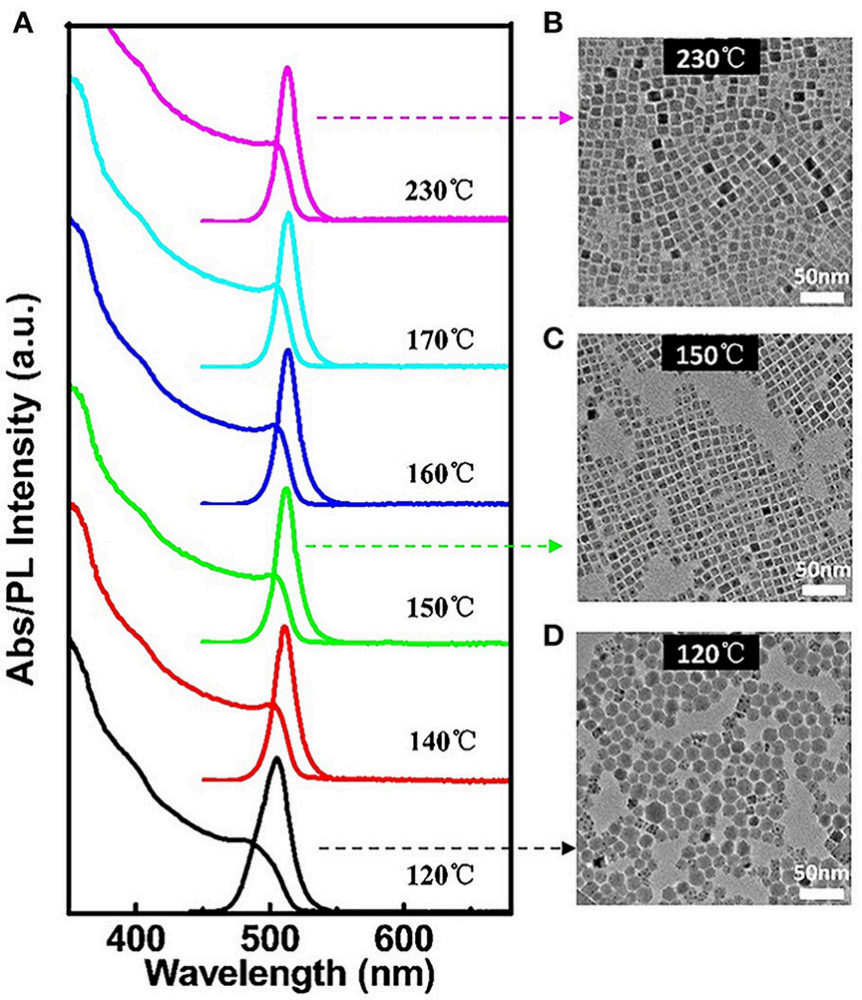
**(A)** UV-visible absorption and PL spectra of CsPbBr_3_ QDs obtained at different injection temperatures after growth for 1 min. TEM images of monodisperse CsPbBr_3_ QDs with an average diameter of: **(B)** 11.8 nm, **(C)** 9.6 nm, and **(D)** 15.0 nm. The corresponding optical spectra are indicated by dashed arrows.

High quality all-inorganic perovskite CsPbBr_3_ QDs are synthesized by injecting PbBr_2_ solution into Cs precursor solution at different temperatures. It is found that the growth temperature plays a crucial role rather than the growth time on the QDs morphology and size, as the nucleation and growth kinetics are very fast, the majority of growth occurs within the first several seconds. Figure 1A shows the UV-visible absorption and PL spectra of CsPbBr_3_ QDs obtained at different injection temperatures. The PL spectrum has a slight redshift with increasing reaction temperatures. As can be seen from the corresponding TEM images of CsPbBr_3_ QDs, hexagonal CsPbBr_3_ QDs are obtained at lower temperature (120°C, Figure 1D). With the increase of temperature over 140°C, monodisperse colloidal nanocubes are synthesized (Figures 1B,C). It is found that the reaction temperature not only influences morphology of CsPbBr_3_ QDs but also influences the PL QYs. For instance, when the reaction temperature is 150°C, the position of PL peak is λ_max_ = 517 nm and the absolute PL QY has maximum value of 90.7%. Nevertheless, when the reaction temperature decreases to 140 and 120°C, the absolute PL QY reduces to 84.9% and 35%, respectively. When the reaction temperature increases to 160 and 170°C, the PL spectra have a redshift to 521 nm and the absolute PL QYs reduce to 69.5 and 62.4%, respectively, and the QY is only about 28% as temperature rises to 230°C. The above results demonstrate that the optimum temperature for the synthesis of the CsPbBr_3_ QDs is 150°C, in which can obtain homogeneous and size-uniformed QDs with high QYs. The corresponding HRTEM image of CsPbBr_3_ QDs synthesized at 150°C is shown in Figure S1, clear lattice fringes indicate the high crystallinity of the sample. The elemental mapping images of CsPbBr_3_ QDs (Figure S2) show that the Cs, Pb, Br elements are dispersed throughout the entire particle. This indicates that the CsPbBr_3_ QDs are uniformly alloyed in the particle rather than form core/shell structures.

**Figure 2:**
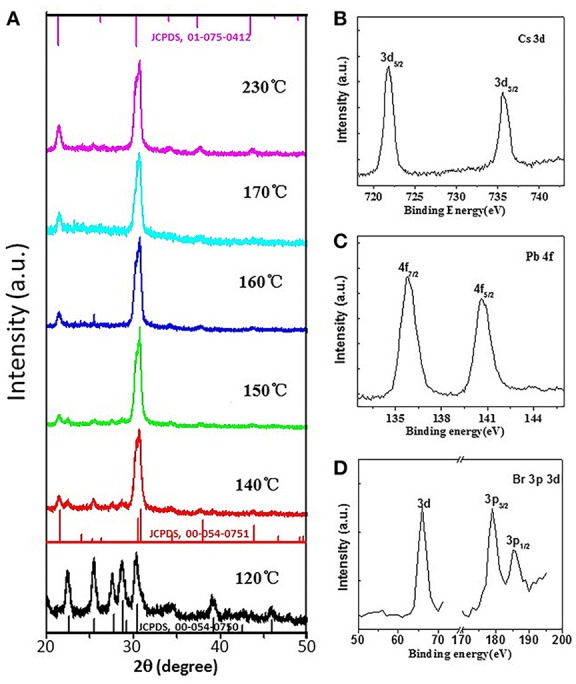
**(A)** XRD patterns of as-synthesized perovskite CsPbBr_3_ QDs formed at different temperatures, **(B–D)** high-resolution XPS spectra of perovskite CsPbBr_3_ QDs formed at 150°C.

To characterize the structures and compositions of CsPbBr_3_ QDs, their crystallographic properties and elements are determined by XRD and XPS (Figure 2A). Generally speaking, the final crystal structure of CsPbX_3_ QDs is strongly dependent on the growth temperature (Li Q. et al., 2017). Higher temperature (≥130°C) generally leads to a cubic phase while lower temperature (<130°C) results in an orthorhombic phase (Ling et al., 2016). XRD patterns of CsPbBr_3_ QDs synthesized at different reaction temperatures are shown in Figure 2A for comparison. It can be seen that orthorhombic phased CsPbBr_3_ QDs (JCPDS, 00-054-0750) are formed when the temperature is at 120°C, whereas cubic CsPbBr_3_ (JCPDS, 01-075-0412) are formed when the temperatures are higher than 130°C. Based on the calculation of the XRD results, the sizes of CsPbBr_3_ QDs are 14.8, 11.3, 10.2, 11.9, 12.2, and 11.9 nm when the reaction temperatures are 120, 140, 150, 160, 170, and 230°C, respectively, which are fit with corresponding TEM results. To further identify the formation of CsPbBr_3_, XPS results are shown in Figures 2B–D. We can see strong peaks which identify the presence of bromine (Br), lead (Pb), cesium (Cs). These results above demonstrate that we have successfully synthesized high quality perovskite CsPbBr_3_ QDs. From the FTIR spectra shown in Figure S3, it is clear that the surface ligands of CsPbBr_3_ QDs are OAm molecules.

**Figure 3:**
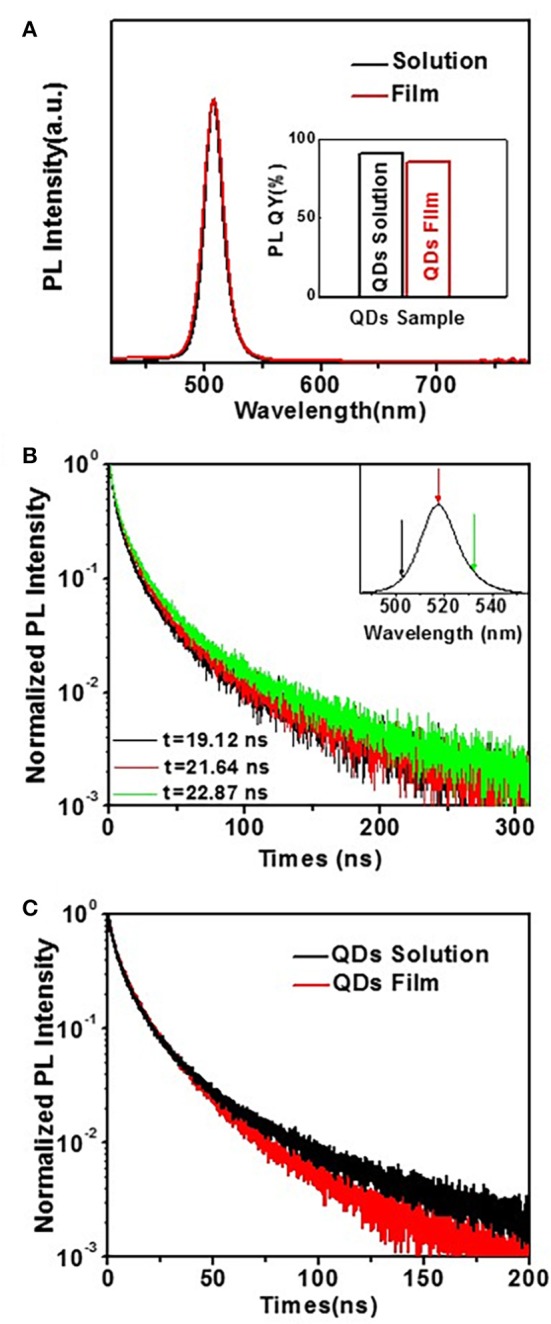
**(A)** PL emission of CsPbBr_3_ QDs (synthesized at 150°C) in the forms of solution vs. solid thin film. Inset: Comparison of PL QYs of CsPbBr_3_ QDs in the forms of solution vs. solid film. **(B)** PL decay curves obtained from perovskite CsPbBr_3_ QDs at different emission wavelengths indicated by the color coded arrows in the insets. **(C)** PL decay curves of diluted hexane solution of perovskite CsPbBr_3_ QDs (black line) vs. films of the QDs layer (red line).

The perovskite CsPbBr_3_ QDs not only possess high PL QYs, but also have only a smaller amount of weakening after forming of film compared with traditional QDs (Swarnkar et al., 2015). CsPbBr_3_ QDs can suppress the self-absorption and FRET after forming of film, which is very beneficial for high device performance of QLED. As shown in Figure 3A, the position of PL peak is λ_max_ = 517 nm for CsPbBr_3_ QDs synthesized at 150°C, with full width at half-maximum (FWHM) of ~18.5 nm. When the QDs are transformed into film, both peak position and relative intensity of CsPbBr_3_ QDs remained almost identical. This characteristic is different from traditional II-VI colloidal QDs. The PL of traditional II-VI colloidal QDs film will have a red-shift relative to the one in solution, which is attributed to (1) higher energy light emitted by a smaller QD is re-absorbed (self-absorption) by a larger QD with a smaller optical gap, and/or (2) the excited smaller QD non-radioactively transfers its energy to a larger QD (FRET) (Li Z. et al., 2017, 2018). The observations may suggest that the self-absorption and FRET are less or they do not influence the PL spectrum of perovskite CsPbBr_3_ film. The inset is absolute PL QYs of QDs solution and film, which reveals that the PL QY has a small reduction from 90.7% in solution to 84.8% in film, which also indicates a low degree of FRET in the case of CsPbBr_3_ QDs film. In order to further verify above discussion, PL decay was measured to investigate the influence of FRET in perovskite CsPbBr_3_ QDs film. Figure 3B shows the PL decay curves obtained from perovskite CsPbBr_3_ QDs at different emission wavelengths, i.e., 500, 517, and 534 nm. The PL decay curves are well-fitted by a biexponential decay fitting, which suggests that the PL decay of CsPbBr_3_ QDs took place through two relaxation pathways: fast decay due to the trap-assisted recombination at grain boundaries and slow decay due to radiative recombination inside the grain (Cho et al., 2015; Kim et al., 2015). With increasing emission energy, there is a slight reduction from 16.7 to 15.6 ns in lifetime, such similar lifetimes at all emission energies suggest less contribution from FRET in CsPbBr_3_ QDs. Figure 3C shows that the lifetime of QDs film is slightly reduced from 16.1 ns in dilute hexane solution to 14.3 ns. This shortening (~88.8%) is far less pronounced compared to that observed in traditional II-VI colloidal QDs, which can further support that the FRET between interacting QD dipoles has less influence on perovskite CsPbBr_3_ QDs film.

**Figure 4:**
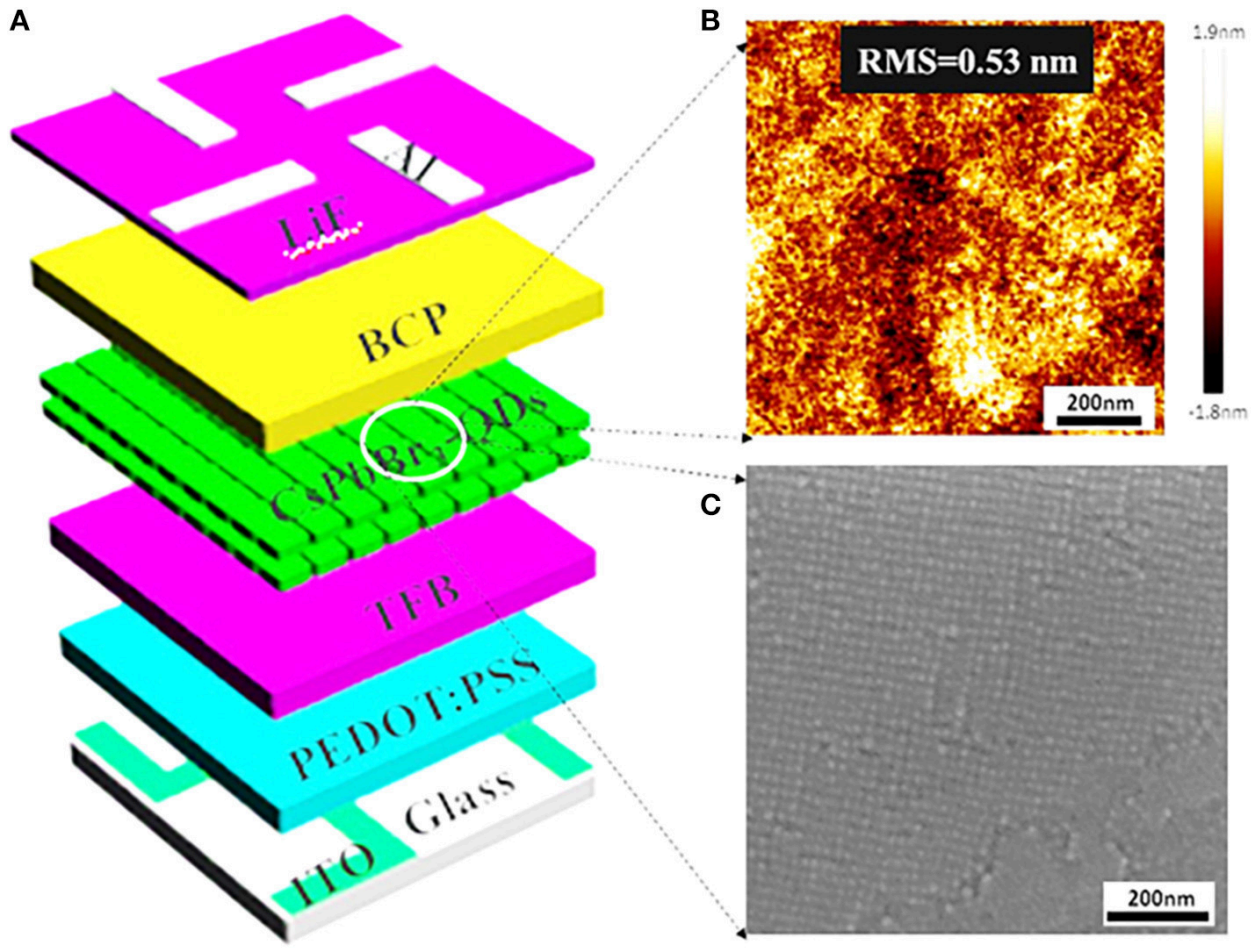
**(A)** Schematic illustration of the QLEDs with a multilayered structure, consisting of ITO/HIL/HTL/perovskite CsPbBr_3_ QDs/BCP/LiF/Al. **(B)** AFM measurement shows the surface roughness of the spin-coated CsPbBr_3_ QDs films (Rq = 0.53 nm) in the device configuration. **(C)** Surface SEM image of uniformly, compactly packed CsPbBr_3_ QDs.

Less FRET and high QY make CsPbBr_3_ QDs huge potential to become a new class of emitting materials in future practical QLEDs. To evaluate the performance of QLEDs based on perovskite CsPbBr_3_ QDs as emitting material, the devices are fabricated according to the structure shown in Figure 4A. The QLEDs with a multilayered structure consists of patterned ITO substrate/PEDOT:PSS/TFB/perovskite CsPbBr_3_ QDs/BCP/LiF/Al. The PEDOT:PSS hole injection layer (HIL), TFB hole transport layer (HTL) and QDs are successively spin-coated on patterned ITO substrate, while BCP, LiF, and Al cathode are deposited successively by thermal vacuum deposition. We use TFB as HTL rather than poly-TPD and PVK, which is because of its high hole mobility and proper HOMO level (−5.3 eV) (Shen et al., 2015). Also, we use CBP as ETL due to the appropriate HOMO (−6.0 eV) and LUMO level (−2.9 eV) of CBP, which is not only in favor of electron injection into the emitting layer, but also provides hole blocking layer. It is different from previous reports that spincoating of MAPbBr_3_ solution often creates a rough, non-uniform surface with many cuboids of large grain size, which may lead to a substantial leakage current and large exciton diffusion length that reduce current efficiency of QLEDs (Cho et al., 2015). Figure 4B presents surface morphologies of CsPbBr_3_ QDs obtained by atomic force microscopy (AFM), indicates that the root mean square roughness (Rq) is less than 1 nm. Relatively uniform surface morphology of spin-deposited, compactly packed QD layer is also observed from scanning electron microscopic (SEM) image in Figure 4C. We attribute the improved uniform surface to the introduction of the TFB layer.

**Figure 5:**
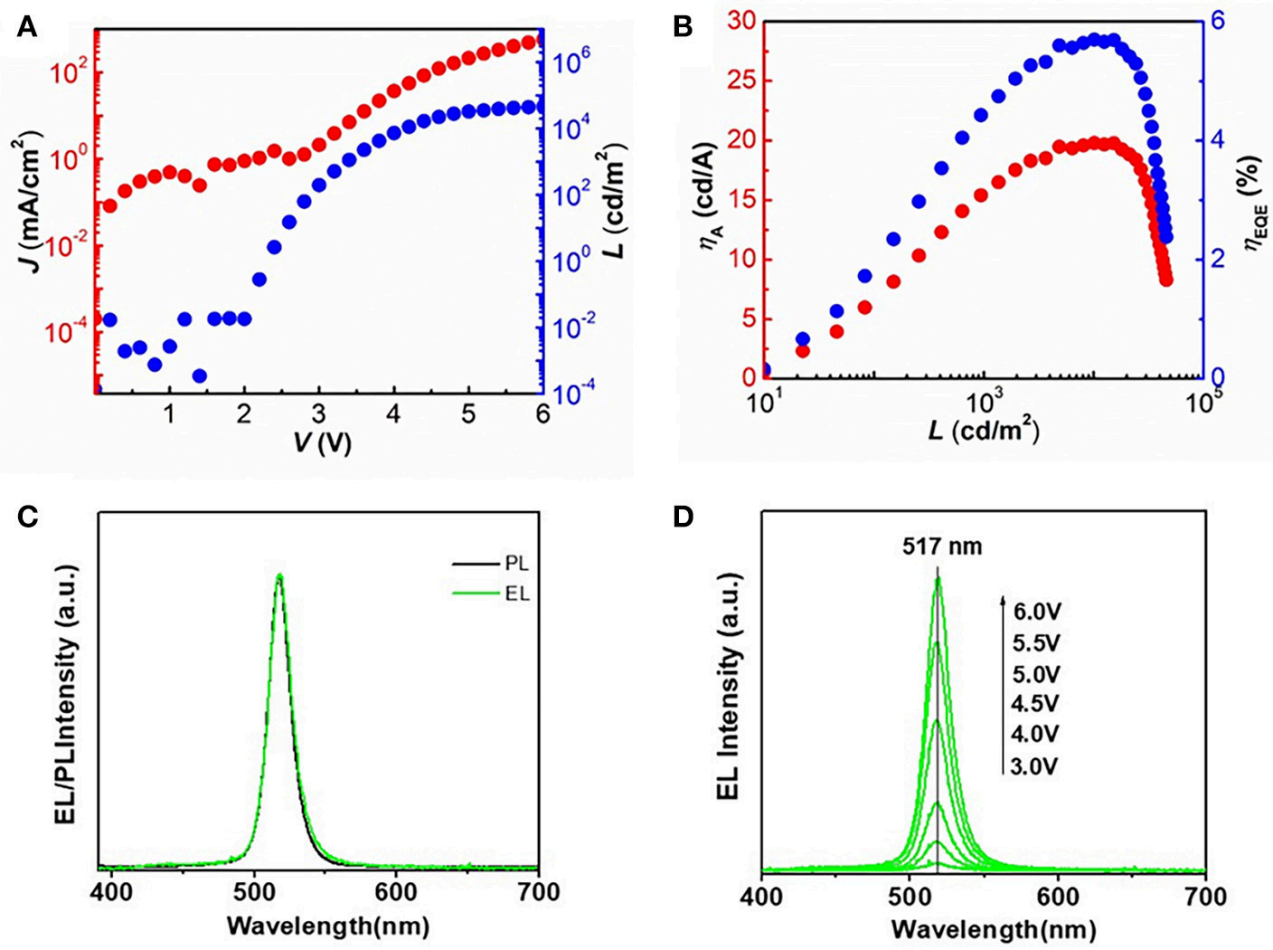
**(A)** Current density (J) and luminance (L) vs. driving voltage characteristics for the optimized QLEDs. **(B)** Current efficiency (η_A_) and EQE as a function of luminance for the optimized QLEDs. **(C)** Normalized PL (black line) and EL spectra (green line) of the QLEDs based on perovskite CsPbBr_3_ QDs. **(D)** Evolution of EL spectra of QLEDs with increasing bias voltage.

Figure 5A shows the current density (J) and luminance (L) vs. driving voltage characteristics for the best CsPbBr_3_ based QLEDs. The QLED based on perovskite CsPbBr_3_ QDs shows a maximum brightness over 46,000 cd/m^2^ (Figure 5A) with a peak EQE and current efficiency of 5.7% and 19.9 cd/A (Figure 5B), respectively, which is among the highest value reported for perovskite CsPbBr_3_ QDs based light-emitting diodes. The significant improvement compared to previous reports is due to the uniform morphology of perovskite film and the excellent structure of QLEDs. The normalized PL and EL spectra of the QLEDs are shown in Figure 5C. The peak position (λ_max_ = 517 nm) and line shape of EL spectrum are identical to that of PL spectrum, which indicates that there is no electric-field-induced Stark effect (Polland et al., 1985). Figure 5D shows the evolution of EL spectra of QLEDs with increasing bias voltage, in which there is no obvious changes in the FWHM (~18.5 nm) and peak position of EL spectra during the testing procedure, which is attributed to the suppression of electric-field-induced Stark effect. These results indicate that carriers are well-confined within the perovskite layers and the recombination zones do not change under different operational conditions, which can ensure good color purity of the device.

## Conclusions

In summary, high quality monoclinic green-emitting CsPbBr_3_ QDs have been successfully synthesized with injecting PbBr_2_ precursors into Cs precursor solution. High brightness with the PL QYs up to 90% and narrow size-distribution with the FWHM below 18.5 nm are obtained under the optimized conditions. Green LEDs based on the CsPbBr_3_ QDs have been successfully demonstrated by using composite films of TFB and BCP layers as the hole-transporting and the electron-transporting layers, respectively. Highly bright green QLEDs showing maximum luminance up to 46,000 cd/m^2^ with a low turn on voltage of 2.3 V, and peak external quantum efficiency (EQE) of 5.7%, corresponding to 19.9 cd/A in luminance efficiency. These devices also show high color purity for electroluminescence (EL) with FWHM <20 nm, and no redshift and broadening with increasing voltage as well as a spectral match between PL and EL.

## Author contributions

HY conducted all experiments in the lab and wrote the first draft of the manuscript. GT and WX performed the LED measurements. SW and HZ performed the quantum dots measurements. JN and XC planned and programmed all experiments and wrote the final manuscript including the discussion.

### Conflict of interest statement

The authors declare that the research was conducted in the absence of any commercial or financial relationships that could be construed as a potential conflict of interest. The reviewer WJ declared a shared affiliation, though no other collaboration, with one of the authors XC to the handling Editor.
