# Supplementary material for: Green Light-Emitting Devices Based on Perovskite CsPbBr3 Quantum Dots
Source: Front Chem. 2018 Aug 28;6:381. doi: 10.3389/fchem.2018.00381 (PMC6151352; doi:10.3389/fchem.2018.00381)
Supplement: Supplementary file 1 [file Table_1.docx]

**Supplementary Material**

**Green Light-emitting Devices Based on Perovskite CsPbBr_3_ Quantum Dots**

**Han Yu^1^, Guimin Tian^2^, Weiwei Xu^2^, Shengwei Wang^2^, Huaikang Zhang^2^, Jinzhong Niu^2^*, Xia Chen^3^**

**^
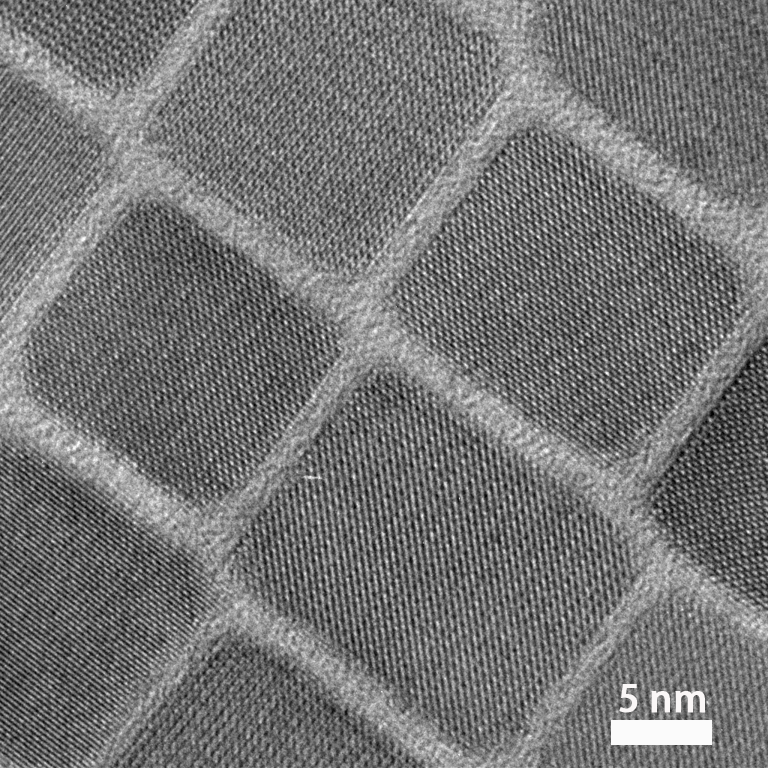
^**

Figure S1. HRTEM image of CsPbBr_3_ QDs synthesized at 150 ºC.


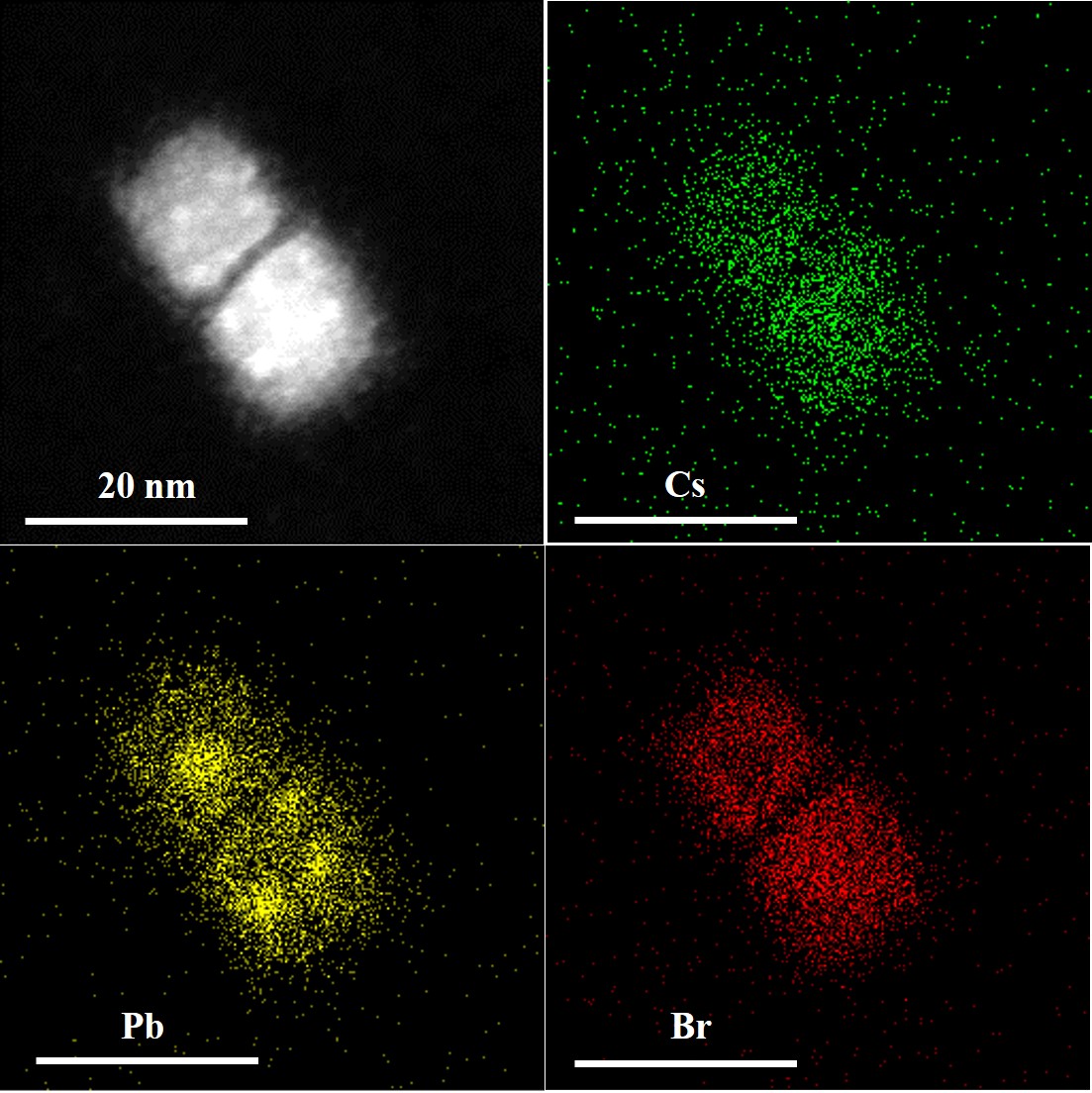


Figure S2. Elemental mapping images of CsPbBr_3_ QDs synthesized at 150 ºC.


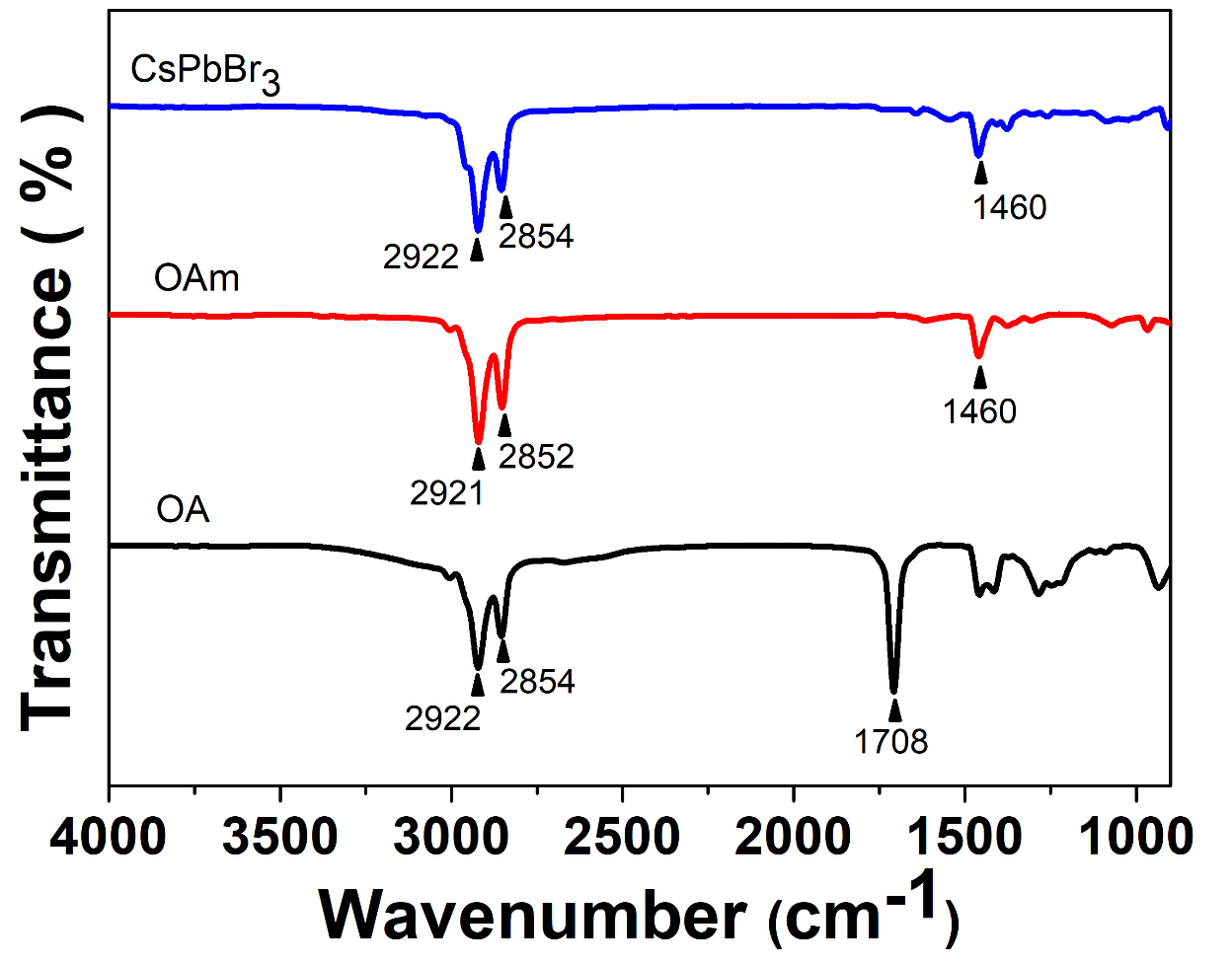


Figure S3. FTIR spectra of OA, OAM, and CsPbBr_3_ QDs synthesized at 150 ºC.
